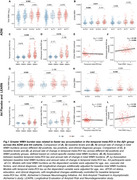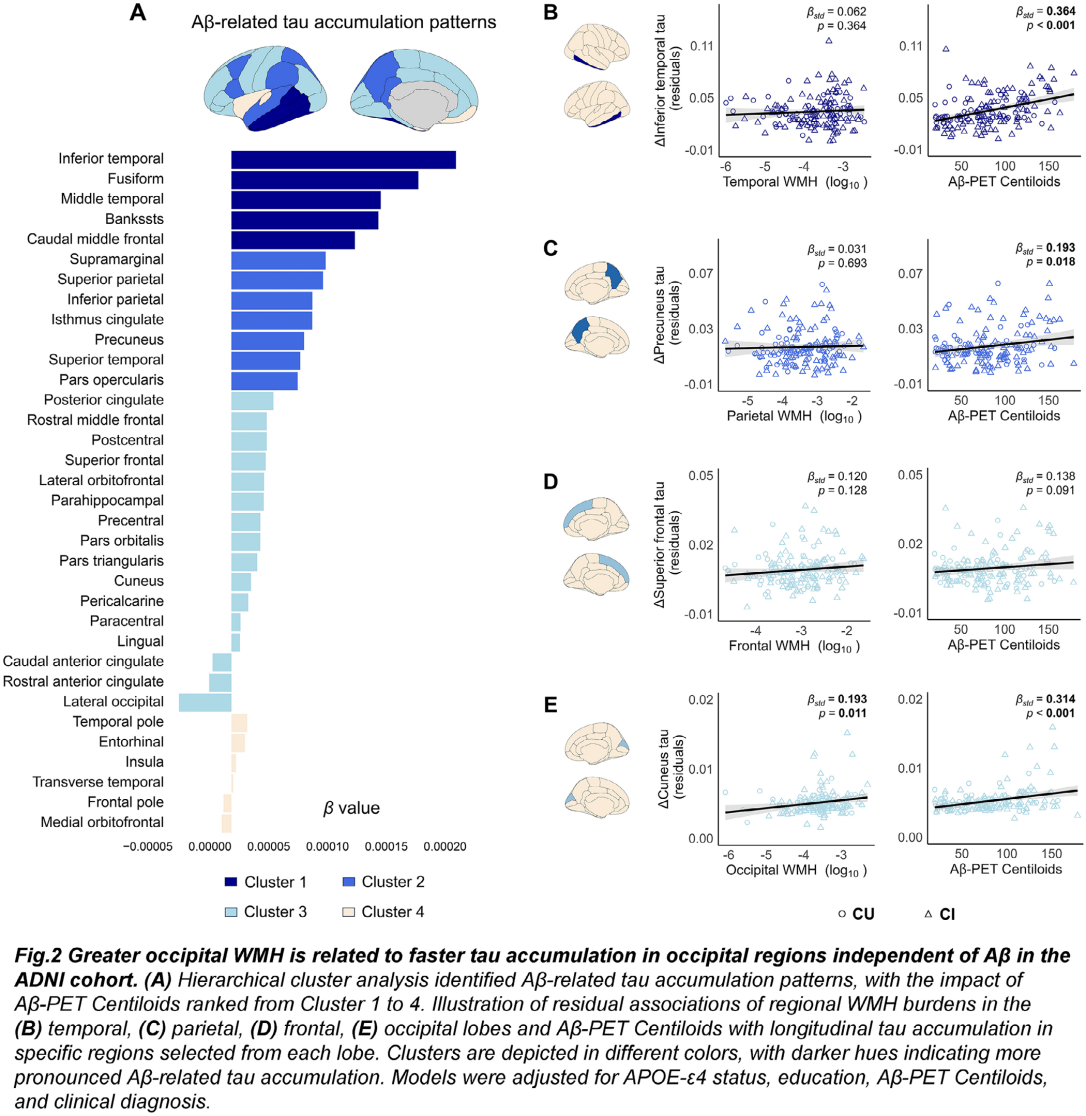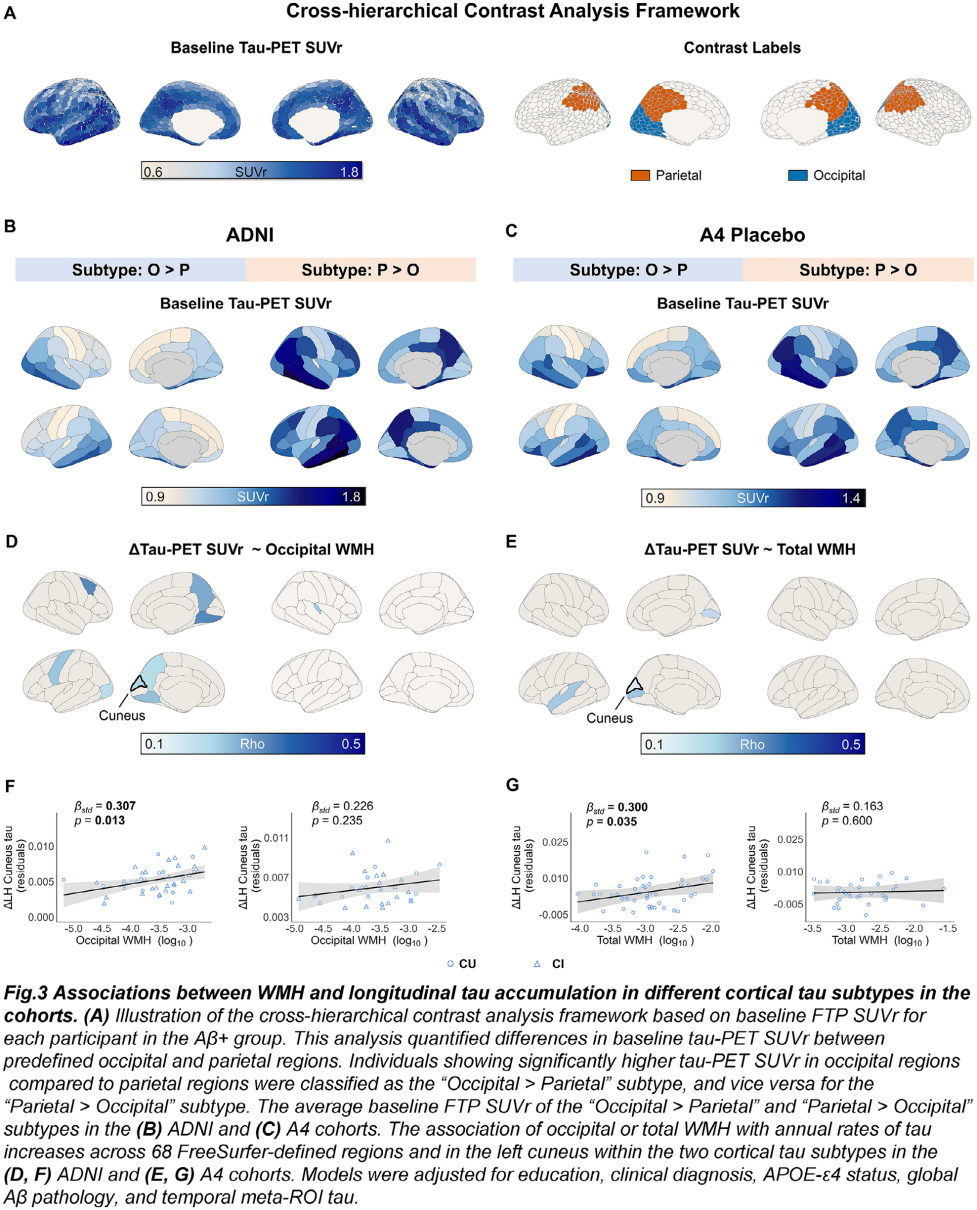# White Matter Hyperintensities Correlate with Accelerated Tau Accumulation in Posterior Cortical Regions in Alzheimer's Disease

**DOI:** 10.1002/alz70856_104870

**Published:** 2026-01-07

**Authors:** Yue Cai, Jie Yang, Li Liang, Lili Fang, Xin Zhou, Anqi Li, Lin Liu, Pan Sun, Zhengbo He, Yalin Zhu, Yiying Wang, Mingxu Li, Ting Ma, Ying Han, Tengfei Guo

**Affiliations:** ^1^ Shenzhen Bay Laboratory, Shenzhen, Guangdong, China; ^2^ Xuanwu Hospital of Capital Medical University, Beijing, Beijing, China; ^3^ Hainan University, Haikou, Hainan, China; ^4^ The Hong Kong University of Science and Technology, Hongkong, Hongkong, China; ^5^ Harbin Institute of Technology, Harbin, Heilongjiang, China; ^6^ The First Affiliated Hospital of Anhui Medical University, Hefei, Anhui, China; ^7^ Harbin Institute of Technology (Shenzhen), Shenzhen, Guangdong, China; ^8^ Peking University Shenzhen Graduate School, Shenzhen, Guangdong, China

## Abstract

**Background:**

White matter hyperintensities (WMH) often co‐exist with β‐amyloid (Aβ) and tau tangles in Alzheimer's disease (AD). However, the association of WMH, Aβ plaques, and tau tangles in AD remains elusive. Using two large datasets, this study comprehensively examined the relationship between regional WMH and longitudinal tau accumulation in AD.

**Method:**

A total of 951 participants from the ADNI and A4 cohorts with Aβ‐PET, fluid‐attenuated inversion recovery images (FLAIR), and tau‐PET data were included, with Resting‐state functional MRI (RS‐fMRI) available for a subset of participants. FLAIR images were segmented using a U‐Net deep learning model to obtain regional WMH volumes. Tau propagation along connectivity patterns was assessed using connectivity‐associated tau spread metrics derived for the whole cortex and specific cortical regions (*β*_Global_, *β*_Frontal_, *β*_Parietal_, *β*_Temporal_, and *β*_Occipital_). We examined the associations between regional WMH, tau accumulation, and connectivity‐associated tau spread. Additionally, two cortical tau subtypes were identified: “Occipital > Parietal” and “Parietal > Occipital”, characterized by higher or lower occipital tau relative to parietal tau, and the impact of regional WMH on tau accumulation was assessed within these subtypes.

**Result:**

Aβ+ individuals showed higher baseline levels and faster increases in total WMH compared to Aβ‐ individuals, but no differences were observed between T+ and T‐ individuals. Among Aβ+ individuals, temporal meta‐ROI tau was not associated with faster WMH increases. However, greater total WMH was linked to accelerated temporal meta‐ROI tau accumulation (Figure 1), although this relationship did not persist after controlling for Aβ. Greater occipital WMH was associated with faster tau accumulation in occipital regions, particularly the cuneus, and with increasing *β*_Occipital_, independent of Aβ (Figure 2). The “Parietal > Occipital” subtype exhibited more rapid tau progression than the “Occipital > Parietal” subtype. In contrast, higher WMH was linked to faster tau increases in the cuneus exclusively within the latter subtype (Figure 3).

**Conclusion:**

Greater WMH burden, particularly in the occipital lobe, is associated with faster tau accumulation and spread in posterior cortical regions, independent of Aβ. These findings provide novel insights into understanding how vascular damages reflected by WMH contribute to cortical tau aggregation in the posterior cortical region of AD.